# Neurotropin Suppresses Inflammatory Cytokine Expression and Cell Death through Suppression of NF-κB and JNK in Hepatocytes

**DOI:** 10.1371/journal.pone.0114071

**Published:** 2014-12-03

**Authors:** Bi Zhang, Yoon Seok Roh, Shuang Liang, Cheng Liu, Mitsuru Naiki, Koichi Masuda, Ekihiro Seki

**Affiliations:** 1 Department of Medicine, School of Medicine, University of California San Diego, La Jolla, California, United States of America; 2 Department of Surgery, School of Medicine, University of California San Diego, La Jolla, California, United States of America; 3 Department of Orthopaedic Surgery, School of Medicine, University of California San Diego, La Jolla, California, United States of America; 4 Department of Pharmacological Research, Institute of Bio-Active Science, Nippon Zoki Pharmaceutical Company Ltd., Kato, Hyogo, Japan; Institute of Hepatology - Birkbeck, University of London, United Kingdom

## Abstract

Inflammatory response and cell death in hepatocytes are hallmarks of chronic liver disease, and, therefore, can be effective therapeutic targets. Neurotropin® (NTP) is a drug widely used in Japan and China to treat chronic pain. Although NTP has been demonstrated to suppress chronic pain through the descending pain inhibitory system, the action mechanism of NTP remains elusive. We hypothesize that NTP functions to suppress inflammatory pathways, thereby attenuating disease progression. In the present study, we investigated whether NTP suppresses inflammatory signaling and cell death pathways induced by interleukin-1β (IL-1β) and tumor necrosis factor-α (TNFα) in hepatocytes. NTP suppressed nuclear factor-κB (NF-κB) activation induced by IL-1β and TNFα assessed by using hepatocytes isolated from NF-κB-green fluorescent protein (GFP) reporter mice and an NF-κB-luciferase reporter system. The expression of NF-κB target genes, *Il6*, *Nos2*, *Cxcl1, ccl5* and *Cxcl2* induced by IL-1β and TNFα was suppressed after NTP treatment. We also found that NTP suppressed the JNK phosphorylation induced by IL-1β and TNFα. Because JNK activation contributes to hepatocyte death, we determined that NTP treatment suppressed hepatocyte death induced by IL-1β and TNFα in combination with actinomycin D. Taken together, our data demonstrate that NTP attenuates IL-1β and TNFα-mediated inflammatory cytokine expression and cell death in hepatocytes through the suppression of NF-κB and JNK. The results from the present study suggest that NTP may become a preventive or therapeutic strategy for alcoholic and non-alcoholic fatty liver disease in which NF-κB and JNK are thought to take part.

## Introduction

Liver fibrosis is caused by the overproduction and deposition of collagen fibers and persistent liver inflammation accompanied by the disabling of normal liver regeneration that is associated with chronic viral hepatitis (e.g., hepatitis B and C), alcoholic liver disease, non-alcoholic steatohepatitis (NASH) and autoimmune hepatitis[1]. Liver cirrhosis is the end result of liver fibrosis, and is developed in virtually all patients with hepatocellular carcinoma (HCC). In the United States, liver cirrhosis is the 12th leading cause of death, with annual deaths of approximately 30 thousand patients[2]. In Japan, the number of deaths by HCC and liver cirrhosis (except for alcoholic liver cirrhosis) is 40 thousand annually, surpassing the mortality of colon cancer patients[3]. Targeting the inhibition of liver inflammation and hepatocyte death can be an effective therapy for chronic liver diseases, including liver fibrosis. However, the discovery, development, and clinical trials for a new drug require enormous research costs and efforts. Therefore, the reassessment of established drugs purposed for other diseases, which have the potential for preventive or therapeutic effects on liver disease, is encouraged.

Neurotropin^®^ (NTP) is a drug derived from a non-protein fraction extracted from the inflamed skin of rabbits after the administration of vaccinia virus. NTP has been used for more than 50 years for treatment of various chronic pain conditions, such as low back pain, cervico-omo-brachial syndrome, post herpetic neuralgia, hyperesthesia of subacute myelo-optic neuropathy (SMON) and other painful conditions and the safety of NTP has already been demonstrated. In experimental animals, Neurotropin shows anti-allodynic and anti-hyperalgesic effects in neuropathic pain models [4], [5], [6], [7], [8], [9], [10]. In addition, the population of patients who are the target of NTP overlaps with the population of patients with chronic liver disease[10], [11], [12].

In chronic liver disease, such as alcoholic and non-alcoholic fatty liver disease, the overproduction of IL-1β and TNFα is observed[13]. IL-1β binds to the IL-1 receptor whose intracellular domain interacts with MyD88 that recruits IRAK4, IRAK1 and TRAF6[14]. Subsequently TAK1 binds to the polyubiquitin chains of TRAF6 to be activated[15]. Activated TAK1 induces the activation of the IKK complex and JNK1. Consequently, the transcriptional factors nuclear factor-κB (NF-κB) and activator protein-1 (AP-1) are activated, which induce the transcription of inflammatory genes, such as *IL6* and *NOS2* in hepatocytes. On the other hand, TNFα binding to trimerized TNF receptor type I forms the complex of the intracellular molecules, TRADD, RIP1 and TRAF2[14]. The TRAF2-linked ubiquitin chains interact with TAK1 to activate TAK1 and its downstream IKK complex and JNK. In addition to the caspase-dependent cell death pathway, TNFα-mediated JNK activation is also associated with the induction of hepatocyte death[14]. We hypothesize that NTP suppresses the IL-1β- and TNFα-mediated inflammatory signaling and cell death pathway through the suppression of NF-κB and JNK activation in hepatocytes, thereby attenuating liver inflammation and hepatocyte damage. Treatment with NTP may become a new approach for chronic liver diseases accompanied by the chronic pain associated with musculo-skeletal disease.

## Materials and Methods

### Mice, Reagents and Cells

Neurotropin® used in this study was provided by Nippon Zoki Pharmaceutical Company Ltd., Osaka, Japan. Wild-type C57BL/6 mice were purchased from the Jackson laboratory (Bar Harbor, ME). The study also used NF-κB-reporter green fluorescent protein (GFP) transgenic mice that express GFP proteins under control of the NF-κB promoter[16]. Primary hepatocytes were isolated from wild-type C57BL/6 mice and NF-κB-reporter GFP transgenic mice by the in situ collagenase perfusion method[17]. Cells with>90% viability were used for the experiments. Two hours after the hepatocytes were plated, M199 medium containing 10% fetal bovine serum (FBS) was changed to serum-free M199 media or 1% FBS M199 media for overnight culture, and subsequently cells were treated as described below. M199 medium was used for hepatocyte culture experiments throughout the study. All mice received humane care according to the National Institutes of Health recommendations outlined in their Guide for the Care and Use of Laboratory Animals. All animal experiments were approved by the UCSD Institutional Animal Care and Use Committee.

### Measurement of NF-κB activation by the GFP reporter

After overnight serum starvation, primary hepatocytes isolated from NF-κB-reporter GFP transgenic mice were first treated with NTP (0.01, 0.1 or 0.2 NU/mL) for 1 hour. The cells were then treated with or without 10 ng/mL IL-1β (R&D Systems, Minneapolis, MN) for 24 hours. The fluorescent signal intensity of GFP of hepatocytes was then measured by fluorescent microscopy.

### NF-κB luciferase assay

After changing the media to 1% FBS M199, wild type primary hepatocytes were infected with adenoviral NF-κB-luciferase reporter at moi 10 for 16 hours[18]. The cells were first treated with NTP (0.2 or 0.4 NU/mL) for 1, 6 or 24 hours before treatment with 2 ng/mL IL-1β or 2 ng/mL TNFα (R&D Systems, Minneapolis, MN). Luciferase activity was measured after 8 hours of the treatment with IL-1β or TNFα. Luciferase activity was normalized to the protein concentration of hepatoctytes in each well.

### Quantitative real-time PCR

Primary hepatocytes were first treated with NTP (0.2 NU/mL) for one hour prior to IL-1β or TNFα treatment, as described above. Two or six hours after treatment with IL-1β or TNFα, Total RNA was extracted using TRIZOL (Life Technologies, Grand Island, NY), followed by reverse transcription of total RNA to cDNA. The cDNA subsequently underwent quantitative real-time polymerase chain reaction (PCR) using the CFX96 real-time PCR system (Bio-Rad, Hercules, CA). PCR primer sequences used were used: *18s* rRNA forward 5′-AGTCCCTGCCCTTTGTACACA-3′. *18s* rRNA reverse 5′-CGATCCGAGGGCCTCACTA-3′. *Il6* forward 5′-ACCAGAGGAAATTTTCAATAGGC-3′. *Il6* reverse 5′-TGATGCACTTGCAGAAAACA-3′. *Nos2* forward 5′-TTCTGTGCTGTCCCAGTGAG-3′. *Nos2* reverse 5′-TGAAGAAAACCCCTTGTGCT-3′. *Ccl5* forward 5′-CCACTTCTTCTCTGGGTTGG-3′. *Ccl5* reverse 5′-GTGCCCACGTCAAGGAGTAT-3′. *Cxcl1* forward 5′-TGCACCCAAACCGAAGTC-3′. *Cxcl1* reverse 5′-GTCAGAAGCCAGCGTTCACC-3′. *Cxcl2* forward 5′-AAAGTTTGCCTTGACCCTGAA-3′. *Cxcl2* reverse 5′-CTCAGACAGCGAGGCACATC-3′. *Junb* forward 5′-CCTGTGTCTGATCCCTGACC-3′. *Junb* reverse 5′-ATCCCTATCGGGGTCTCAAG-3′. Gene expression was normalized to 18s RNA as an internal control.

### Western blot

Protein extracts were electrophoresed, blotted, and then incubated with antibodies for phospho-JNK, phospho-p65 (Ser536), caspase-3, cleaved caspase-3 (Cell Signaling, Danvers, MA), IκBα, p65, JNK (Santa Cruz Biotechnology, Dallas, TX), and β-actin (Sigma-Aldrich, St. Louis, MO) with appropriate secondary horseradish peroxidase (HRP)-conjugated antibodies, and developed.

### Immunofluorescence

Primary hepatocytes were pretreated with NTP (0.2 NU/mL) for 1 hour, and then treated with 2 ng/mL IL-1β or 2 ng/mL TNFα for 15 min. Then cells were fixed and incubated with antibody to p65 (Santa Cruz Biotechnology) and DAPI, and imaged with fluorescent microscopy. Cells with p65-positive nucleus were counted in 8 high power fields (x200).

### Assessment of hepatocyte death

After cell attachment, hepatocytes were serum-starved for 16 hours, and first treated with NTP (0.2 NU/mL) for one hour. The cells were then treated with Actinomycin D (200 ng/mL; Sigma-Aldrich, St. Louis, MO) and IL-1β (2 ng/mL) or TNFα (2 ng/mL) for an additional 16 hours [17]. Apoptosis was examined by using the TUNEL staining kit (Roche, Indianapolis, IN). TUNEL positive cells were counted in 10 low power fields (x100).

### Statistics

Differences between two groups were compared using the two-tailed unpaired student t-test. Differences between multiple groups were compared using one-way ANOVA using GraphPad Prism 4.02 (GraphPad Software, La Jolla, CA). P values <0.05 were considered significant. All experiments were performed at least three times and the representative data were presented.

## Results

### NTP suppresses NF-κB activation in hepatocytes

**Figure 1:**
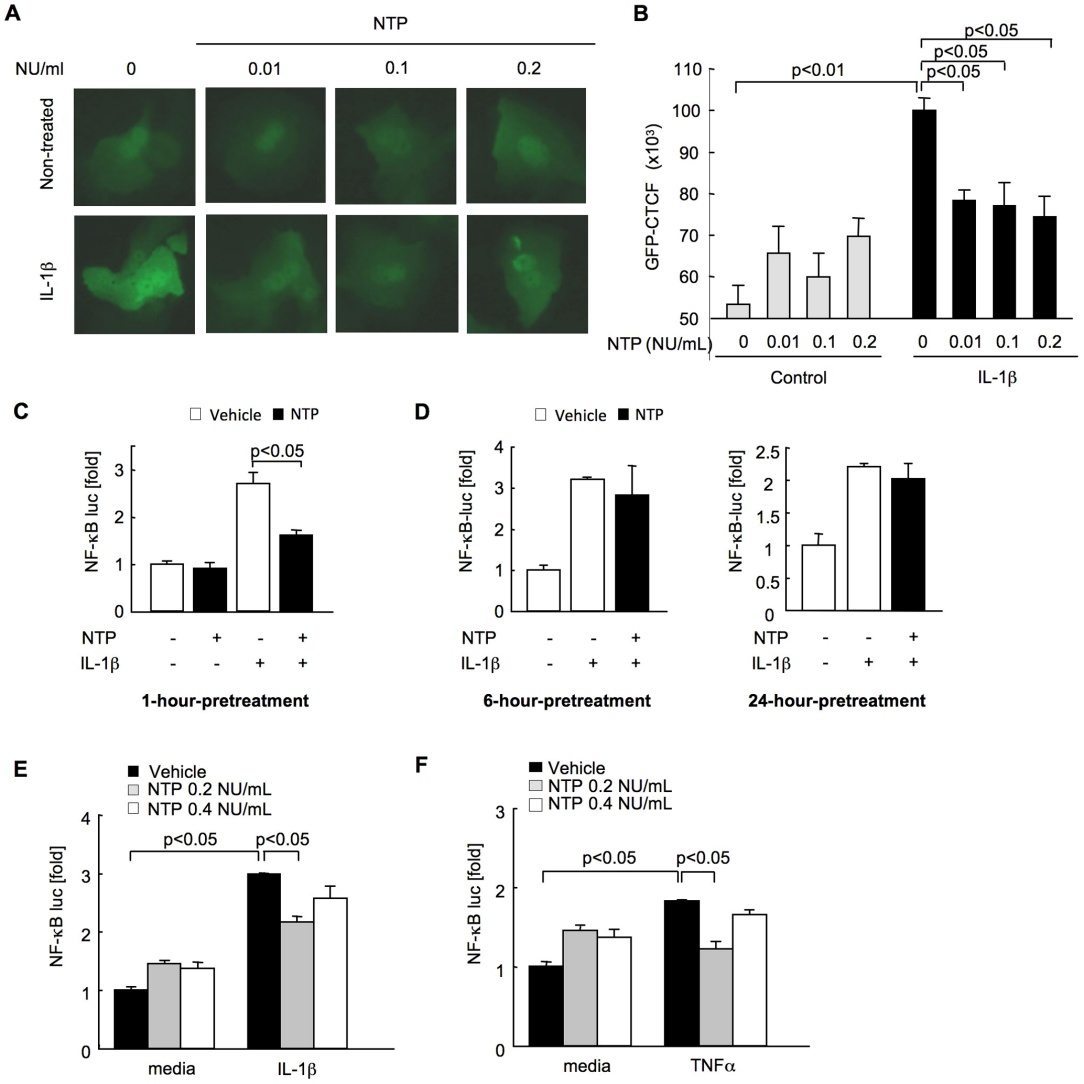
The suppression of interleukin-1β (IL-1β) and tumor necrosis factor-α (TNFα)-induced nuclear factor-κB (NF-κB) activation by pretreatment of hepatocytes with Neurotropin (NTP). **(A, B)** Primary hepatocytes were isolated from NF-κB-GFP reporter transgenic mice. One hour after pretreatment with NTP (0.01, 0.1 and 0.2 NU/mL), hepatocytes were treated with 10 ng/mL IL-1β for 24 hours followed by fluorescent microscopy. Representative pictures are shown (A). Fluorescence of GFP intensity was measured (B). **(C-F)** Wild type (WT) primary hepatocytes were infected with adenoviral NF-κB-luciferase reporter for 16 hours. **(C, D)** Subsequently, cells were pretreated with 0.2 NU/mL NTP for 1 (C), 6 or 24 hours (D) followed by treatment with 2 ng/mL IL-1β for 8 hours and then luciferase activity was measured. **(E, F)** Cells were pretreated with 0.2 or 0.4 NU/mL NTP for 1 hour followed by treatment with 2 ng/mL IL-1β (E) or TNFα (F) for 8 hours and then luciferase activity was measured. Data represent the mean±SEM of triplicate cultures. A representative result is shown. Similar results were obtained in three independent experiments.

**Figure 2:**
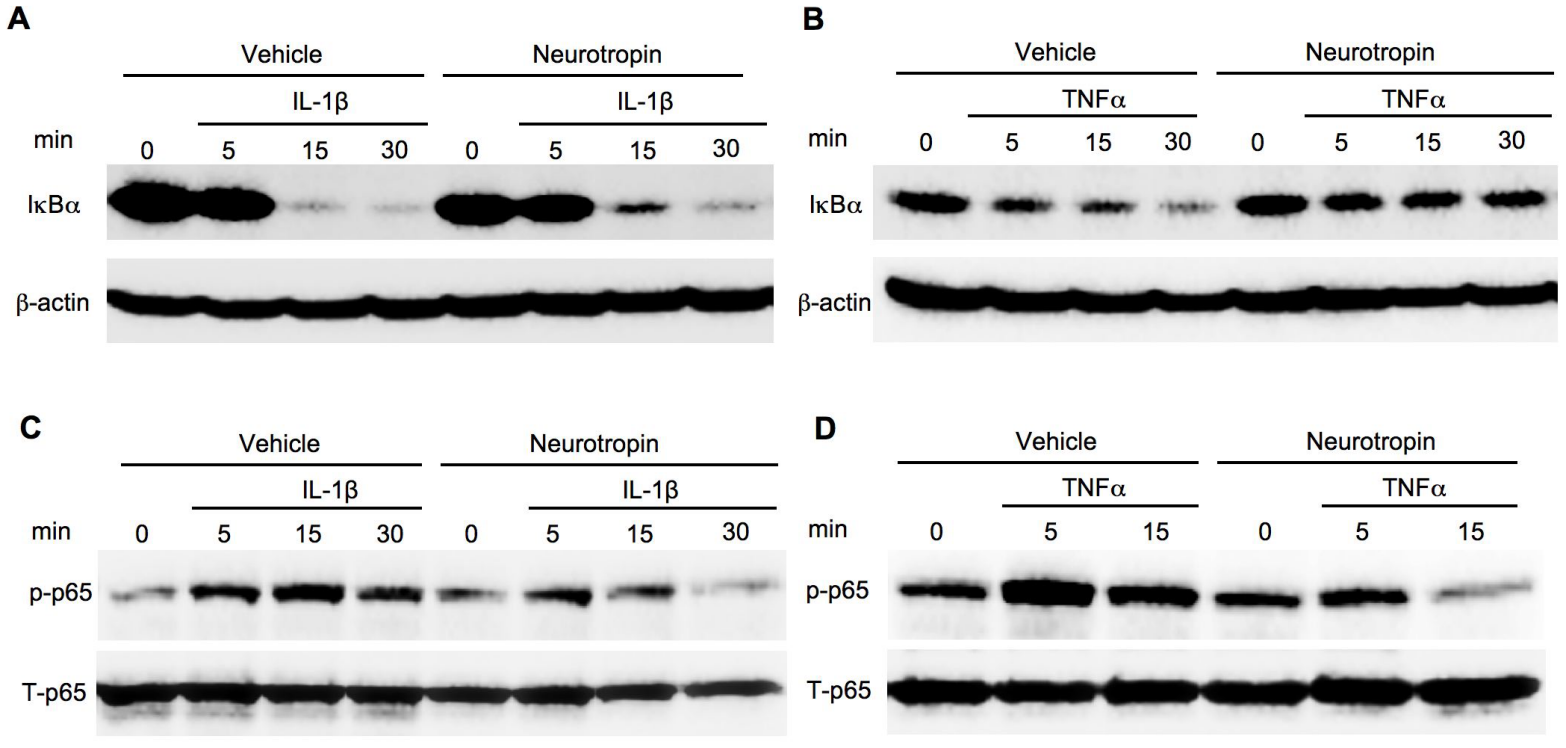
Activation of the nuclear factor-κB (NF-κB) pathway induced by interleukin-1β (IL-1β) and tumor necrosis factor-α (TNFα) is suppressed by pretreatment with Neurotropin (NTP). **(A-C)** Wild type (WT) primary hepatocytes were pretreated with 0.2 NU/mL NTP for 1 hour followed by the treatment with 2 ng/mL IL-1β (A, C) or TNFα (B, D) for 5, 15, or 30 minutes. Western blots for IκB (A, B), phospho-NF-κBp65 (C, D), total p65 and β-actin are shown. A representative result is shown. Similar results were obtained in three independent experiments.

**Figure 3:**
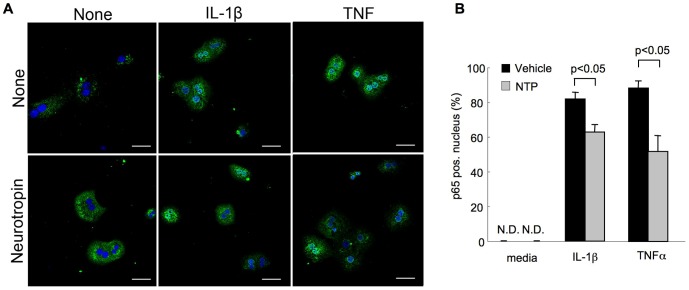
Neurotropin (NTP) suppresses interleukin-1β (IL-1β) and tumor necrosis factor-α (TNFα)-induced nuclear translocation of the nuclear factor-κB (NF-κB). Wild type (WT) primary hepatocytes were pretreated with 0.2 NU/mL NTP for 1 hour followed by the treatment with 2 ng/mL TNFα or IL-1β for 15 minutes. Immunofluorescence for NF-κBp65 is shown. Representative staining for p65 (A; Green, p65; Blue, Nucleus) and quantification (B) are shown. Scale bar, 40 µm. Data represent the mean±SEM of 8 high power fields (x200).

It has been reported that over-activation of NF-κB in hepatocytes is associated with sustained liver inflammation [19]. Because IL-1β is a major activator of NF-κB in hepatocytes and a potent driver of liver inflammation [20], [21], we investigated the effect of NTP on NF-κB activity in hepatocytes. To take advantage of the expression of GFP protein induced by activated NF-κB in NF-κB GFP reporter mice, we used primary hepatocytes isolated from these mice [16]. IL-1β treatment significantly increased NF-κB activity (p<0.01) in hepatocytes as quantified by measuring GFP fluorescent signal intensity (Figure 1A,B). NTP treatments at 0.01, 0.1 and 0.2 NU/ml one hour prior to IL-1β treatment significantly suppressed the IL-1β-induced NF-κB activation (p<0.05) (Figure 1A,B). We then investigated NF-κB activity by using the NF-κB luciferase reporter system. We also assessed the effect of different durations and concentrations of NTP pretreatment on NF-κB activation. We found that a suppressive effect of NTP on IL-1β-mediated NF-κB activity was seen in cells with pretreatment for one hour (p<0.05), but not for 6 or 24 hours (Figure 1C, D). In addition, we found that 0.2 NU/mL is the most effective concentration for the suppressive effect of NTP on NF-κB activation induced by IL-1β and TNFα (Figure 1E, F). These results indicate that NTP at 0.2 NU/mL can suppress the NF-κB activation induced by IL-1β and TNFα in hepatocytes. However, we found that pretreatment with 0.4 NU/mL NTP slightly elevated the NF-κB activity compared with the 0.2 NU/mL NTP pretreatment, suggesting that the higher concentration of NTP may have a stimulatory effect, rather than a suppressive effect on NF-κB activation. We also examined IκB degradation and phosphorylation of NF-κBp65 to further confirm the preventive effect of NTP on NF-κB activation. IL-1β and TNFα rapidly induced IκBα degradation (Figure 2A, B). NTP treatment caused delayed IκBα degradation induced by IL-1β and TNFα (Figure 2A, B), which indicates that NF-κB activation is suppressed by NTP treatment. Phosphorylation of p65 was observed from 5 min to 15 min after treatment with IL-1β and TNFα, whereas these phosphorylations were suppressed by NTP pretreatment (Figure 2C, D). Nuclear translocation of p65 was also seen in hepatocytes treated with IL-1β and TNFα (Figure 3). The NTP pretreatment suppressed IL-1β and TNFα-induced p65 nuclear translocation (Figure 3).

### Proinflammatory gene expression is suppressed by NTP in hepatocytes

**Figure 4:**
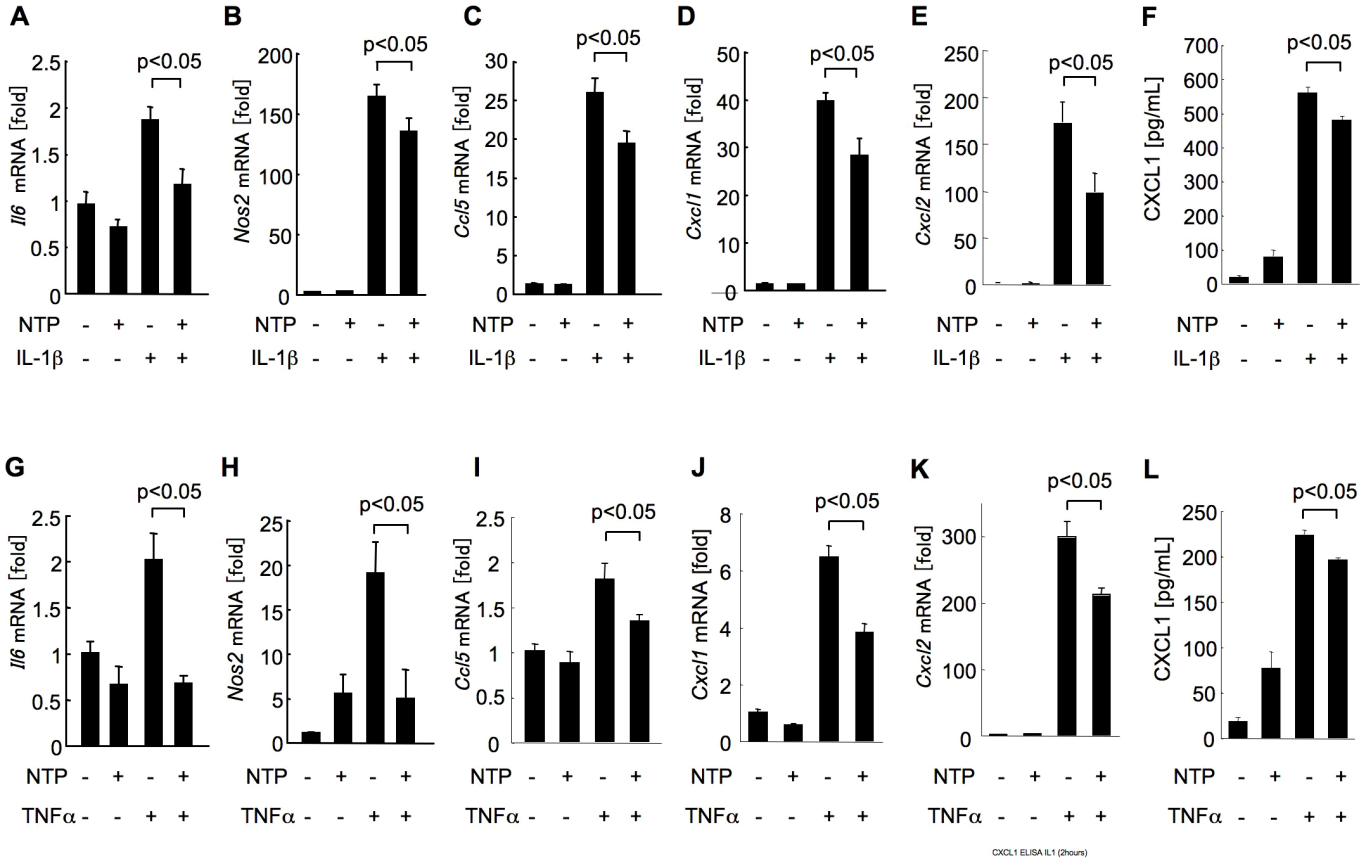
Pretreatment with Neurotropin (NTP) suppresses the gene expression of inflammatory mediators by hepatocytes. **(A-L)** Wild type (WT) primary hepatocytes were pretreated with 0.2 NU/mL NTP for 1 hour followed by treatment with 2 ng/mL interleukin-1β (IL-1β) (A-F) or tumor necrosis factor-α (TNFα) (G-L) for 2 (D,E,J,K) or 6 hours (A-C, G-I). The mRNA expression of *Il6*, *Nos2*, *Ccl5*, *Cxcl1* and *Cxcl2* was measured by quantitative real time PCR (A-E, G-K). The protein levels of CXCL1 secreted to supernatant were measured by ELISA (F, L). Data represent the mean±SEM of triplicate cultures. A representative result is shown. Similar results were obtained in three independent experiments.

Because NF-κB induces an inflammatory response in the liver, we next investigated whether NTP can suppress the induction of inflammatory mediators in hepatocytes. IL-1β treatment induced the upregulation of mRNA expression of *Il6*, *Nos2*, *Ccl5*, *Cxcl1* and *Cxcl2* (Figure 3A-E). The NTP treatment significantly (p<0.05) attenuated the expression of IL-1β-induced *Il6*, *Nos2*, *Ccl5*, *Cxcl1* and *Cxcl2* in hepatocytes (Figure 4A-E). TNFα also induced the increase of mRNA expression of *Il6*, *Nos2*, *Ccl5*, *Cxcl1* and *Cxcl2*, which was significantly suppressed (p<0.05) by NTP pretreatment (Figure 4G-K). CXCL1 protein secreted into the supernatant was increased by treatment with IL-1β and TNFα (Figure 4F, L). The NTP treatment partially but significantly suppressed CXCL1 production induced by IL-1β and TNFα (Figure 4F, L). These results demonstrated that NTP has the capacity to prevent the inflammatory cytokine production mediated by IL-1β and TNFα in hepatocytes.

### IL-1β and TNFα-mediated JNK activation is reduced by NTP pretreatment

**Figure 5:**
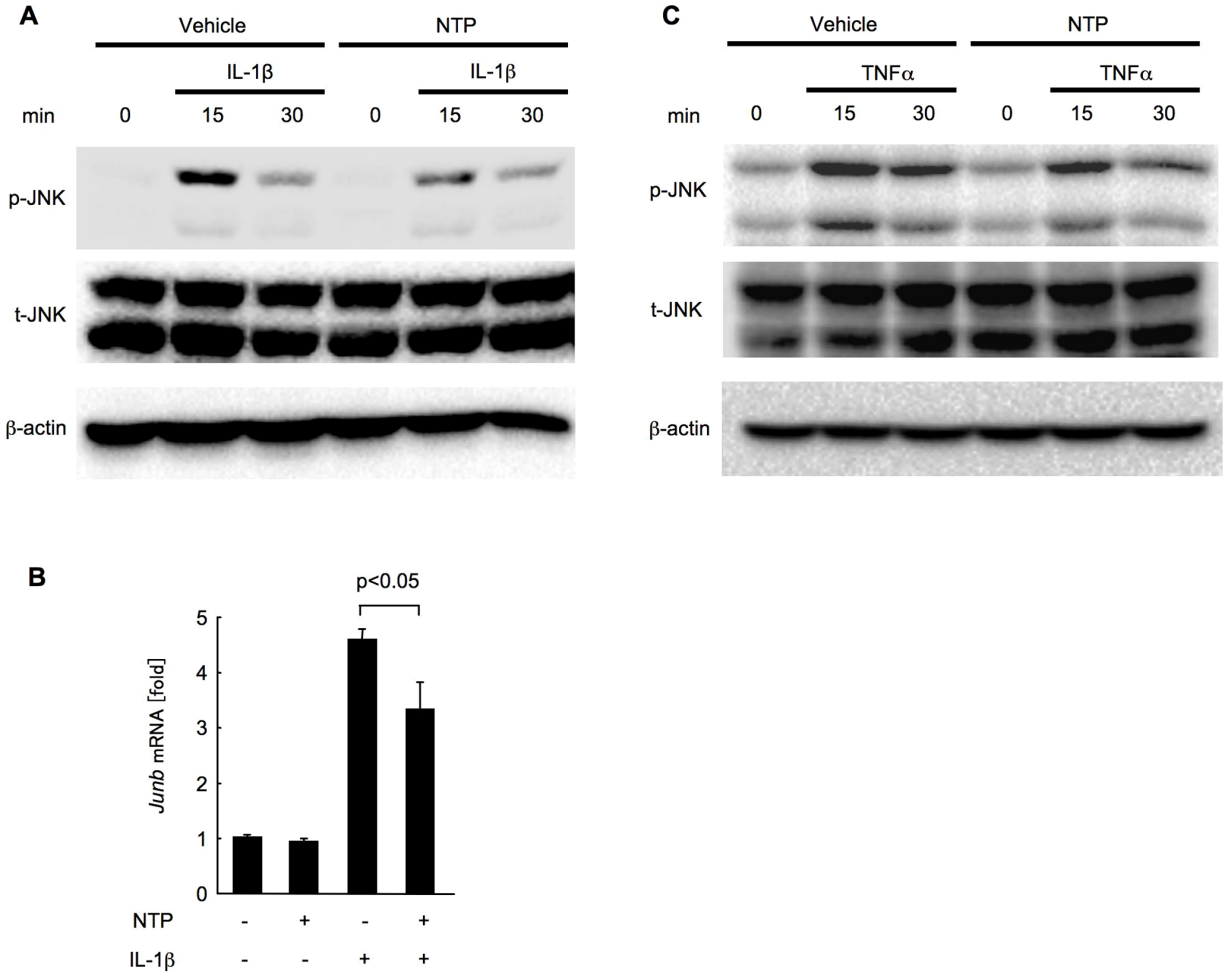
Pretreatment with Neurotropin (NTP) suppresses interleukin-1β (IL-1β) and tumor necrosis factor-α (TNFα)-induced JNK activation in hepatocytes. (A-C) Wild type (WT) primary hepatocytes were pretreated with 0.2 NU/mL NTP for 1 hour followed by the treatment with 2 ng/mL IL-1β (A, B) or TNFα (C) for 15 or 30 minutes (A, C), or two hours (B). Western blots for phospho-JNK, total JNK and β-actin are shown (A, C). Representative blots are shown. Similar results were obtained in three independent experiments. mRNA expression of *Junb* was measured by quantitative real time PCR. Data represent the mean±SEM of triplicate cultures.

In addition to NF-κB, JNK is also activated by IL-1β and TNFα in hepatocytes [14]. We therefore examined the effect of NTP on JNK activation. Upon IL-1β treatment, JNK was quickly phosphorylated in hepatocytes (Figure 5A). The IL-1β-mediated JNK activation was reduced by NTP treatment (Figure 5A). Furthermore, the upregulation of the expression of the JNK target gene *Junb* by IL-1β was significantly suppressed (Figure 5B). Similarly, TNFα induced an immediate activation of JNK in hepatocytes, and the TNFα-induced JNK phosphorylation was suppressed by NTP pretreatment (Figure 5C). These findings indicate that NTP pretreatment reduces not only NF-κB, but also JNK activation in hepatocytes.

### NTP pretreatment suppresses IL-1β-mediated hepatocyte death

**Figure 6:**
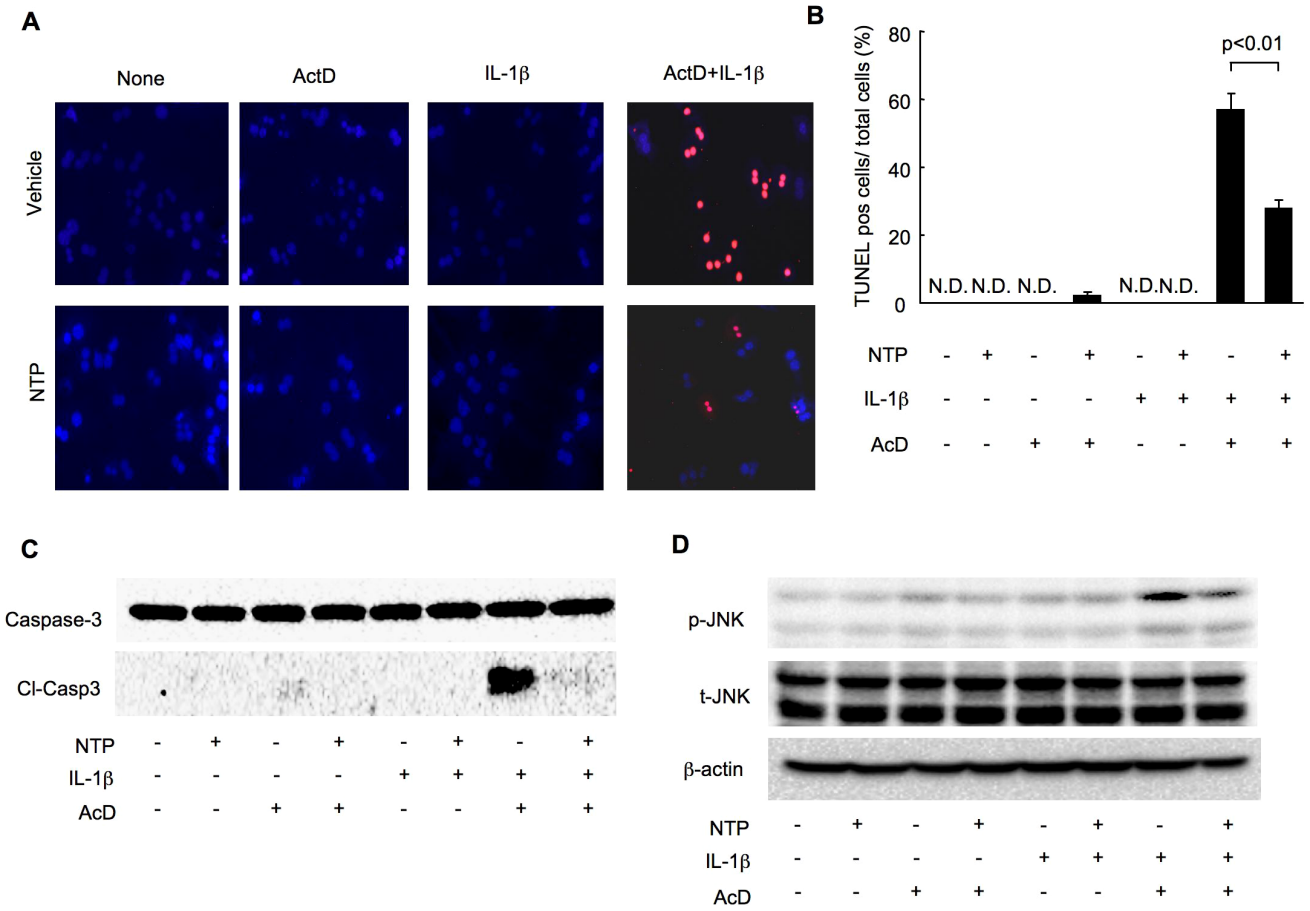
Interleukin-1β (IL-1β)-mediated hepatocyte death is attenuated by pretreatment with Neurotropin (NTP). **(A-D)** Wild type (WT) primary hepatocytes were pretreated with 0.2 NU/mL NTP for 1 hour followed by the treatment with 2 ng/mL IL-1β with or without 200 ng/mL Actinomycin D for 16 (A, B), 8 (C) or 4 hours (D). Representative TUNEL staining (A; Red, TUNEL positivity; Blue, Nucleus.) and quantification (B) are shown. Data represent the mean±SEM of triplicate cultures. Western blots for caspase-3, cleaved caspase-3 (C), phospho-JNK, total JNK and β-actin are shown (D). Representative blots are shown. Similar results were obtained in three independent experiments.

We found that NTP suppresses JNK activation that is known to promote hepatocyte death [14]. We therefore examined the potential of NTP to prevent hepatocyte death. Because IL-1β alone does not induce hepatocyte death[20], hepatocytes were treated with and without sensitization by Actinomycin D during treatment with IL-1β. In combination with Actinomycin D, IL-1β treatment caused evident hepatocyte death after 16 hours of treatment (Figure 6A, B). Of note, NTP treatment showed a marked reduction of IL-1β plus Actinomycin D-induced hepatocyte death as assessed by TUNEL staining (Figure 6A, B). Cleaved caspase-3 levels were also reduced by NTP treatment (Figure 6C). Consistently, increased JNK activation by IL-1β plus Actinomycin D was suppressed by NTP treatment (Figure 6D). These results indicate that NTP can prevent hepatocyte death mediated by IL-1β.

### TNFα and Actinomycin D-induced hepatocyte death is prevented by NTP pretreatment

**Figure 7:**
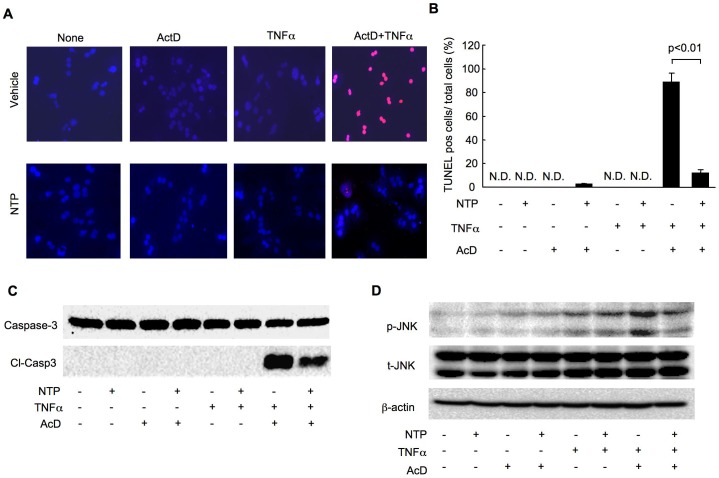
Tumor necrosis factor-α (TNFα)-mediated hepatocyte death is suppressed by pretreatment with Neurotropin (NTP). **(A-D)** Wild type (WT) primary hepatocytes were pretreated with 0.2 NU/mL NTP for 1 hour followed by the treatment with 2 ng/mL TNFα with or without 200 ng/mL Actinomycin D for 16 (A, B) 8 (C) or 4 hours (D). Representative TUNEL staining (A; Red, TUNEL staining; Blue, Nucleus) and quantification (B) are shown. Data represent the mean±SEM of triplicate cultures. Western blots for caspase-3, cleaved caspase-3 (C), phospho-JNK, total JNK and β-actin are shown (D). Representative blots are shown. Similar results were obtained in three independent experiments.

In addition to IL-1β, we tested the preventive effect of NTP on TNFα-mediated hepatocyte death. Similar to IL-1β, TNFα alone failed to induce hepatocyte death, whereas sensitization with Actinomycin D during treatment with TNFα caused remarkable hepatocyte death after 16 hours (Figure 7A, B). TUNEL staining showed that NTP treatment dramatically reduced hepatocyte death caused by treatment with TNFα plus Actinomycin D (Figure 7A, B). Cleaved caspase-3 levels were consistently suppressed by NTP treatment (Figure 7C). Additionally, JNK activation in hepatocytes treated with TNFα plus Actinomycin D was significantly attenuated by treatment with NTP (Figure 7D).

## Discussion

Although the therapeutic effect of NTP on chronic pain that is associated with musculo-skeletal disease has been established for decades, the effect of NTP on liver disease has not been extensively investigated[10], [11], [12]. Chronic local inflammation is possibly associated with chronic pain through production of inflammatory cytokines, including IL-1β and TNFα [22], [23], [24]. It is suggested that the analgesic effect of NTP might be mediated through suppression of inflammatory cytokines and their signaling. Given the anti-inflammatory potential of NTP, NTP may have preventive or therapeutic effects on chronic liver inflammation, which is further associated with liver fibrosis. The present study demonstrated that NTP suppressed both NF-κB and JNK signaling pathways induced by IL-1β and TNFα through an unknown mechanism. Furthermore, we confirmed that the expression of the NF-κB-target genes, *Il6*, *Nos2, Cxcl1* and *Cxcl2,* and the AP-1 target gene *Junb*, was also suppressed by pretreatment with NTP. It is known that NF-κB has dual roles in hepatocytes. IL-1β and TNFα are factors that promote alcoholic liver disease and NASH through NF-κB activation[25]. Moreover, hepatocyte-specific IKKβ transgenic mice, in which hepatocyte NF-κB is over-activated, exhibited spontaneous liver inflammation with evident liver fibrosis[19]. Thus, over-activation of NF-κB can promote liver inflammation, and the inhibition of exacerbated NF-κB activation may attenuate liver inflammation. On the other hand, NF-κB is associated with the induction of anti-apoptotic genes, such as *Bcl2* and *Bclxl*, that inhibit caspase-dependent and JNK-dependent hepatocyte apoptosis[25]. In fact, IL-1β or TNFα alone does not cause hepatocyte death[20], [26]. However, inhibition of NF-κB by an IκB super-repressor or inhibition of transcription by Actinomycin D, sensitizes hepatocytes to IL-1β and TNFα-mediated death[26]. Thus, the excessive inhibition of NF-κB enhances liver injury. Our data demonstrated that NTP suppressed IL-1β and TNFα-induced NF-κB activation, but did not suppress these to basal levels, suggesting that the magnitude of the inhibitory effect caused by NTP may only have preventive or therapeutic potential, but may not cause detrimental hepatocyte damage. The inhibition of hepatocyte-derived inflammatory mediators, including IL-6, iNOS and chemokines, may further prevent liver inflammation.

In contrast to the dual roles of NF-κB, the JNK pathway is associated with the promotion of liver inflammation and hepatocyte death[14]. Persistent liver inflammation causes sustained JNK activation that increases the generation of reactive oxygen species and further activates JNK in the liver. Moreover, the sustained JNK activation further activates the E3 ubiquitin ligase Itch that ubiquitinates and degrades c-FLIP, an endogenous caspase-8 inhibitor[14]. This results in caspase-8 activation leading to hepatocyte apoptosis[14]. In addition, JNK activation induces the translocation of the proapoptotic proteins, *BAX* and *BID*, to mitochondria to promote mitochondria-mediated caspase-9 activation, thereby inducing hepatocyte death[14]. Our data show that NTP pretreatment inhibited IL-1β and TNFα-mediated JNK activation. The NTP treatment consistently prevented IL-1β and TNFα-mediated death of hepatocytes elicited by Actinomycin D. These results indicate that NTP can prevent liver inflammation and hepatocyte death through suppression of JNK activation induced by IL-1β and TNFα. Sustained hepatocyte damage also suppresses normal liver regeneration, which is one of the factors for the aberrant regenerative response that includes liver fibrosis. It is conceivable that the suppression of persistent hepatocyte damage by NTP may restore the normal regenerative capacity of the liver. The effect of NTP on hepatic regeneration is currently under investigation. The results from the present study prompted us to hypothesize the preventive or therapeutic potential of NTP on alcoholic liver disease and non-alcoholic fatty liver disease in which IL-1β and TNFα play an important role in disease progression. Further investigation is required to test this hypothesis.

Our data suggest that the optimal dose of NTP is important for therapeutic use. The 0.2 NU/mL NTP dose showed an inhibitory effect on IL-1β-induced NF-κB activation, but the inhibitory effect of NTP was weaker in conditions with the 0.4 NU/mL dosage. Thus, NTP may have an adverse effect at higher doses in hepatocytes. Given the narrow range and presumably short half-life of NTP, further study is required for the determination of appropriate therapeutic dose of NTP.

Importantly, most anti-inflammatory drugs, such as non-steroidal anti-inflammatory drugs (NSAIDs), are associated with detrimental effects on the liver. Therefore, the development or discovery of anti-inflammatory drugs with beneficial effects, or without any adverse effects, on the liver is highly attractive. The present study demonstrated two valuable attributes of NTP that have been unrecognized. First, NTP has an anti-inflammatory property. In particular, NTP can block the activities of proinflammatory NF-κB and JNK. Second, NTP may have a preventive or therapeutic potential for chronic liver disease. In the context of these results, we need further investigations. First, the mechanism by which NTP prevents NF-κB and JNK activation induced by IL-1β and TNFα needs to be investigated. We speculate that NTP may suppress the molecule(s) shared between IL-1β and TNFα signaling. However, it is unclear whether NTP suppresses activation of NF-κB and JNK signaling separately or suppresses molecule(s) shared for activation of NF-κB and JNK, such as TAK1 or K63 polyubiquitination chains of TRAFs [15]. Second, because NTP contains various different small molecules including nucleic acids, amino acids and sugars [10], the components of NTP responsible for its inhibitory effect on proinflammatory signaling should be elucidated. Third, the effect of NTP on other liver cells, such as Kupffer cells, endothelial cells and hepatic stellate cells, should be investigated. Fourth, we need to test the preventive or therapeutic effects of NTP on preclinical animal models of chronic liver disease, such as alcoholic liver disease, NASH and liver fibrosis. Thus, future investigations are required to determine whether NTP can be a new therapeutic agent for chronic liver disease, in particular, alcoholic and non-alcoholic fatty liver disease, in which NF-κB and JNK are thought to take part.
